# Identification of Ferroptosis-Related Molecular Clusters and Immune Characterization in Autism Spectrum Disorder

**DOI:** 10.3389/fgene.2022.911119

**Published:** 2022-08-11

**Authors:** Lichun Liu, Yongxing Lai, Zhidong Zhan, Qingxian Fu, Yuelian Jiang

**Affiliations:** ^1^ Department of Pharmacy, Fujian Children’s Hospital, Fuzhou, China; ^2^ Department of Geriatric Medicine, Shengli Clinical Medical College of Fujian Medical University, Fuzhou, China; ^3^ Department of Pediatric Intensive Care Unit, Fujian Children’s Hospital, Fuzhou, China; ^4^ Department of Pediatric Endocrinology, Fujian Children’s Hospital, Fuzhou, China

**Keywords:** autism spectrum disorder, ferroptosis, molecular clusters, immune characteristics, predictive model

## Abstract

**Introduction:** Autism spectrum disorder (ASD) is a neurodevelopmental disorder with clinical presentation and prognostic heterogeneity. Ferroptosis is a regulated non-apoptotic cell death program implicated in the occurrence and progression of various diseases. Therefore, we aimed to explore ferroptosis-related molecular subtypes in ASD and further illustrate the potential mechanism.

**Methods:** A total of 201 normal samples and 293 ASD samples were obtained from the Gene Expression Omnibus (GEO) database. We used the unsupervised clustering analysis to identify the molecular subtypes based on ferroptosis-related genes (FRGs) and evaluate the immune characteristics between ferroptosis subtypes. Ferroptosis signatures were identified using the least absolute shrinkage and selection operator regression (LASSO) and recursive feature elimination for support vector machines (SVM-RFE) machine learning algorithms. The ferroptosis scores based on seven selected genes were constructed to evaluate the ferroptosis characteristics of ASD.

**Results:** We identified 16 differentially expressed FRGs in ASD children compared with controls. Two distinct molecular clusters associated with ferroptosis were identified in ASD. Analysis of immune infiltration revealed immune heterogeneity between the two clusters. Cluster2, characterized by a higher immune score and a larger number of infiltrated immune cells, exhibited a stronger immune response and was markedly enriched in immune response-related signaling pathways. Additionally, the ferroptosis scores model was capable of predicting ASD subtypes and immunity. Higher levels of ferroptosis scores were associated with immune activation, as seen in Cluster2. Lower ferroptosis scores were accompanied by relative immune downregulation, as seen in Cluster1.

**Conclusion:** Our study systematically elucidated the intricate correlation between ferroptosis and ASD and provided a promising ferroptosis score model to predict the molecular clusters and immune infiltration cell profiles of children with ASD.

## Introduction

Autism spectrum disorder (ASD) is a multifactorial disorder that occurs in children and is manifested as a lack of social interaction, activities, and interests and repetitive stereotypical patterns of behaviors (Lord et al., 2018). At present, approximately one to two percent of children in the United States suffer from ASD, and the incidence of ASD is increasing each year (Tordjman et al., 2018). The pathogenesis and underlying molecular mechanisms of ASD have been not yet fully understood. Furthermore, it is widely recognized that the heterogeneity of prognosis and clinical symptoms in children with ASD may be the vital factor contributing to the limited treatment outcome (Gyawali and Patra, 2019; Alpert, 2021). Therefore, further exploration of the pathogenesis and accurate differentiation of ASD subtypes at molecular levels could provide an important theoretical basis for developing individualized treatment strategies for children with ASD.

Unlike other forms of regulated cell death (RCD) such as apoptosis, necrosis, autophagy, and pyroptosis, ferroptosis is recognized as a novel mode of cell death triggered by Fe^2+^ -dependent lipid peroxidation, and with distinct biological properties and pathophysiological processes (Xie et al., 2016; Gyawali and Patra, 2019). Under stressful conditions, intracellular Fe^2+^ overload causes the excessive accumulation of free radicals represented by reactive oxygen species (ROS), which, in turn, promotes the generation of lipid peroxides by combining with Fe^2+^ through the Fenton reaction, thereby leading to an imbalance in the intracellular oxidation–antioxidation system and the corruption of DNA, proteins and other molecules (Toyokuni et al., 2017; Shen et al., 2018; Bjørklund et al., 2020). In addition, cells undergoing ferroptosis usually present remarkable impairment of mitochondrial morphology, as evidenced by mitochondrial shrinking, the decline of mitochondrial volume, disruption of mitochondrial cristae and membrane structure, and enhanced membrane density (DeHart et al., 2018; Wang et al., 2020). It has been reported that the activation of ferroptosis is mainly involved in the occurrence and development of cancer, degenerative and cardiovascular diseases, and stroke (Stockwell et al., 2017; Shin et al., 2018; Wu et al., 2021; Zhang et al., 2021). For example, CDO1 promotes ROS production and lipid peroxidation by blocking GSH synthesis, ultimately inducing ferroptosis, which is closely related to its inhibition of cysteine metabolism (Hao et al., 2017). In addition, suppressing GSH and GPX4 expression levels is usually accompanied by the accumulation of Fe^2+^ and the activation of lipid peroxidation in Alzheimer’s disease (AD) (Raven et al., 2013). A recent study has identified four key ferroptosis-related genes (FRGs) and constructed a diagnostic model for ASD based on these FRGs (Wu X. et al., 2022). In addition, another study found that selenium could enhance autism-relevant behaviors and constrain ASD mouse induced ferroptosis (Wu H. et al., 2022). Nonetheless, the ferroptosis-related molecular subtypes and molecular mechanisms in ASD remain to be illustrated. Therefore, elucidating the correlation between FRGs and the progression of ASD and identifying the molecular clusters based on FRGs may help clarify the ASD heterogeneity at a molecular level.

In the present study, we systematically identified the differentially expressed FRGs and immune infiltration differences between normal subjects and ASD patients. Based on the expression profiles of 16 differential genes related to ferroptosis, a total of 293 ASD children were classified into two ferroptosis-related subgroups, and the biological functions, enriched pathways, and immune profiles between the two clusters were further explored. In addition, we established a scoring system called ferroptosis scores to disclose individual ferroptosis levels. The nomogram and calibration curves indicated that the ferroptosis scores model is a prospective marker for predicting ASD subtypes.

## Materials

### Data Acquisition and Processing

**FIGURE 1:**
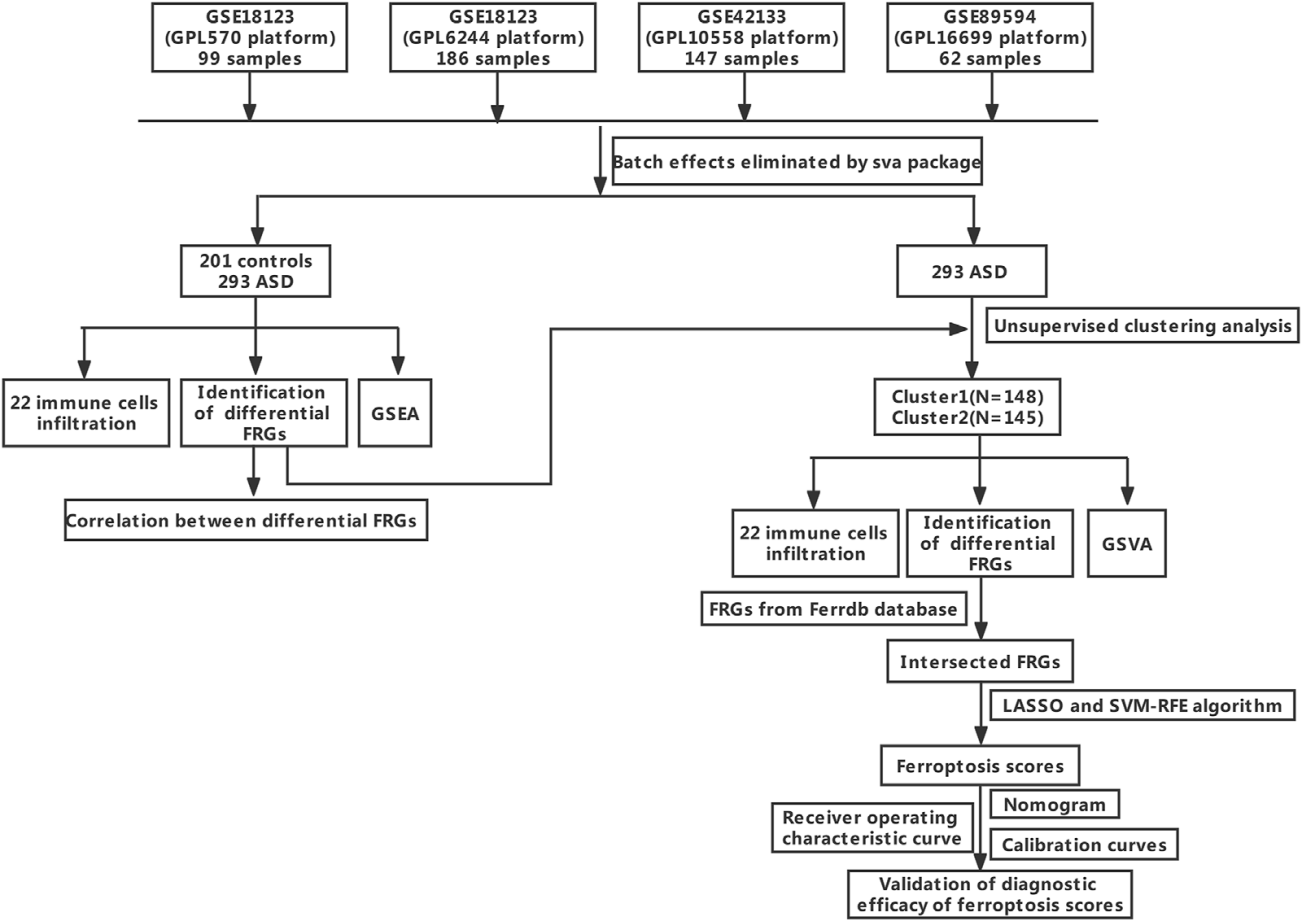
The study flow chart.

Three GEO datasets (GSE18123, GSE42133, and GSE89594) associated with ASD were obtained from the Gene Expression Omnibus (GEO, www.ncbi.nlm.nih.gov/geo) website database using the R package of GEOquery (version 2.60, www.bioconductor.org/packages/2.6/bioc/html/GEOquery.html) (Davis and Meltzer, 2007). A total of 201 non-ASD healthy subjects and 293 ASD children were analyzed. The sample information for each dataset is exhibited in Table 1. The comprehensive flowchart of our study is illuminated in Figure 1.

**TABLE1:** Information for ASD-related datasets.

GO accession	Platform	Samples		Sample source	Age (months or years)		Sex (male/female)	
		Control	ASD		Control	ASD	Control	ASD
GSE18123	GPL570	33	66	Peripheral blood	108.03 ± 5.7	95.77 ± 3.98	33/0	66/0
GSE18123	GPL6947	82	104	Peripheral blood	96.44 ± 5.61	97.10 ± 4.77	48/34	80/24
GSE42133	GPL10558	56	91	Peripheral blood	--	--	56/0	91/0
GSE89594	GPL16699	30	32	Whole blood	23.93 ± 0.41	23.97 ± 0.80	15/15	16/16

The GSE18123 dataset (platform GPL570) consisted of peripheral blood samples of 33 control subjects and 66 ASD children. The GSE18123 dataset (platform GPL6244) included peripheral blood samples of 82 control subjects and 104 ASD children. The GSE42133 dataset (platform GPL10558) was composed of the peripheral blood from 56 healthy subjects and 91 ASD children. The GSE89594 dataset (platform GPL16699) originated from peripheral blood, which included 30 control subjects and 32 ASD children.

These three original datasets underwent background adjustment using the limma R package, and the batch effect of the original gene expression landscapes in these three datasets was eliminated using the comBat function based on the R package of “sva” (version 3.40, http://www.bioconductor.org/packages/3.4/bioc/html/sva.html) (Ritchie et al., 2015). Principal component analysis (PCA) was used to estimate the performance of the comBat algorithm.

### Unsupervised Clustering of Ferroptosis-Related Genes

Initially, A total of 259 FRGs were downloaded from the FerrDb online database (http://www.zhounan.org/ferrdb/). Afterward, 16 FRGs were identified as the differentially expressed genes (DEGs) between control and ASD samples. Then, according to the expression level of these 16 FRGs, an unsupervised clustering analysis was carried out to classify 293 ASD cases into distinct clusters by using the R package of ConsensusClusterPlus (version 2.60, https://www.bioconductor.org/packages/2.6/bioc/html/ConsensusClusterPlus.html) (Wilkerson and Hayes, 2010). The optimal number of classifications was determined by the cumulative distribution function (CDF) curves, consistency clustering score of each cluster and consensus clustering plot.

### Functional and Pathway Enrichment Analysis

The R package of GSVA (version 2.11, https://www.bioconductor.org/packages/2.11/bioc/html/GSVA.html) was used for enrichment analysis, which assesses the distinct pathway’s activity and biological functions between different ferroptosis clusters (Hänzelmann et al., 2013). The “c2.cp.kegg.v7.4.symbols.gmt” and “c5.go.bp.v7.5.1.symbols.gmt” files were obtained from the MSigDB online database (http://www.gsea-msigdb.org/gsea/msigdb) for GSVA enrichment analysis. The adjusted *p*-value of less than 0.05 was determined as the significantly enriched pathways and biological functions.

The R packages of msigdbr (version 7.50.1, https://cran.r-project.org/web/packages/msigdbr/index.html) and GSEABase (version 2.11, https://www.bioconductor.org/packages/2.11/bioc/html/GSEABase.html) were applied for Gene Set Enrichment Analysis (GSEA) between control and ASD group. The C2:GO:BP and C2: CP:KEGG gene sets were utilized as reference gene sets, and those with a value of *p* less than 0.05 were defined as significantly enriched gene sets.

### Analysis of Differentially Expressed Gene Related to Ferroptosis Subtypes

The DEGs between different ferroptosis subtypes were determined using the R package of limma (version 3.52.1, http://www.bioconductor.org/packages/release/bioc/html/limma.html). Genes with an adjusted *p*-value less than 0.001 were considered to be statistically significant. The ggplot2 R package (version 3.3.5, http://had.co.nz/ggplot2/) was utilized to plot the volcano plot, and the R package of pheatmap (version 1.0.12, https://cran.r-project.org/web/packages/pheatmap/index.html) was applied for drawing the heatmap of DEGs.

### Immune Cell Infiltration Analysis

CIBERSORT algorithm (https:/cibersort.stanford.edu/) was utilized to estimate the abundance of 22 immune cell subtypes, which was performed on R software (Newman et al., 2019). The LM22.txt file containing the characteristics of gene expression matrix based on 22 immune cell subtypes and the gene expression matrix containing 293 ASD samples served as the input file of the “CIBERSORT” algorithm, and the estimated composition proportion of the 22 immune cell subtypes in each sample was subsequently visualized. Finally, we used the estimate (version 1.0.13, https://bioinformatics.mdanderson.org/estimate/rpackage.html) R package to calculate the immune score of each ASD patient. A *p*-value less than 0.05 was considered statistically significant.

### Co-Expression Network Construction by Weighted Gene Co-Expression Network Analysis

The R package of WGCNA (version 1.71, https://cran.r-project.org/web/packages/WGCNA/index.html) was used for constructing the co-expression network of all genes in the 293 ASD samples (Langfelder and Horvath, 2008). The optimal power value was selected to transform the gene expression matrix into a weighted adjacency matrix, which was further transformed into a topological overlap matrix (TOM). Then, the hierarchical clustering algorithm was utilized to cut the cluster tree structure of highly co-expressed genes to create different modules. The module eigengene (ME) contained the whole expression level of the gene in each module. Modular significance (MS) represented the correlation between different gene modules and disease traits. Gene significance (GS) was applied for exhibiting the correlation between relative module and module membership.

### Construction of the Ferroptosis Scores Model

A total of 28 FRGs were identified by intersecting the DEGs screened by the DEGs and WGCNA algorithm with the ferroptosis genes obtained from the FerrDb database. The 293 ASD samples were randomly classified into training cohort (70%) and validation cohort (30%). The LASSO model was constructed on the basis of expression profiles of these FRGs by using the “glmnet” (version 4.1.3, https://cran.r-project.org/web/packages/glmnet/index.html) R package (Gao et al., 2010). The hub genes were determined based on the optimal lambda value. In order to illustrate the diagnostic value of the LASSO model to distinguish ASD subtypes, the AUC values of receiver operating characteristic (ROC) curves were plotted in the training set and validation set by using the R package pROC (version 1.18.0, https://cran.r-project.org/web/packages/pROC/index.html). The support vector machine–recursive feature elimination (SVM-RFE) machine learning algorithm with linear kernel was performed to acquire the optimal variables *via* 10-fold cross-validation using the R package of “caret” (version 6.0.92, https://cran.r-project.org/web/packages/e1071/index.html). Linear SVM allocated correspond weights (w) to each feature variable, and RFE was utilized to select optimal feature subsets *via* recursively screening smaller and smaller feature subsets in the training set. RFE algorithm was performed using the RFE function in the caret package, and the accuracy was applied for evaluating the performance of a distinct number of feature variables. Subsequently, the optimal number of feature variables that exhibited the highest overall accuracy were utilized as the input variables to reconstruct the liner-SVM model using the SVM function in the caret package. Afterward, the reconstructed model was utilized to predict the training set and validation set, respectively. The performance of the SVM model in the training and testing sets was evaluated using the AUC values of receiver operating characteristic (ROC) curves based on the R package pROC. The final key FRGs were determined by intersecting the results of these two machine learning algorithms, and their correlative coefficients were utilized to calculate ferroptosis scores as follows: ferroptosis scores = ∑_I_ Coefficients_i_ × Expression level of gene_i_.

### Statistics

The difference between the two groups was analyzed using the Wilcoxon test. Spearman correlation test was carried out to analyze the gene–gene correlation by using the “psych” (version 2.2.5, https://cran.r-project.org/web/packages/psych/index.html) package. All the statistical tests conducted were two-sided, and a value of *p* < 0.05 was considered to be statistically significant.

## Results

### Dysregulation of FRGs and Activation of the Immune System in Autism Spectrum Disorder Patients

**FIGURE 2:**
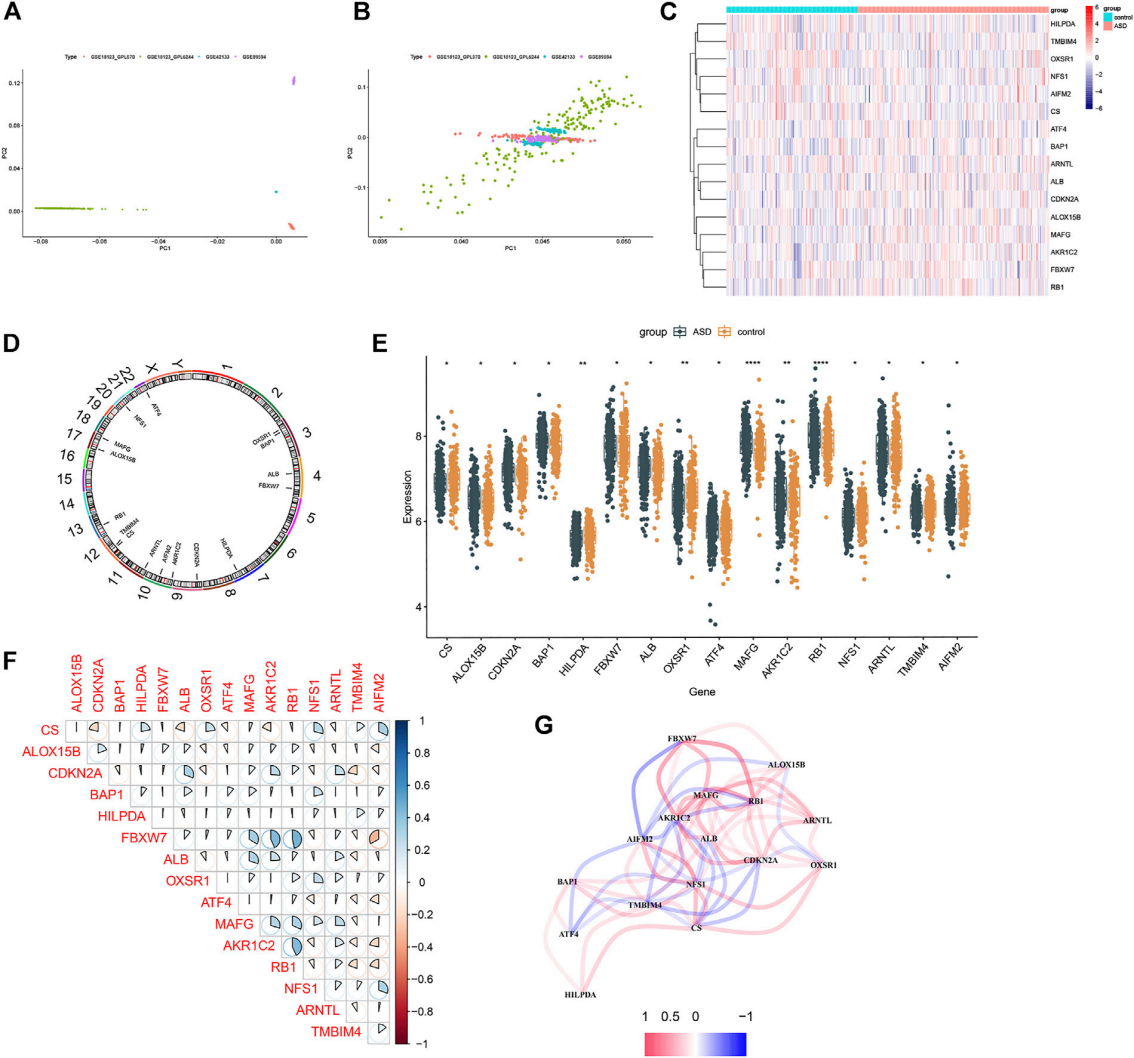
Differential FRGs screening in ASD children. **(A** and **B)** Principal component analysis of different datasets before **(A)** and after **(B)** batch correction. **(C)** Representative heatmap of 16 differentially expressed FRGs. **(D)** The location of 16 differentially expressed ferroptosis genes on chromosomes. **(E)** Box plots showing the expression of 16 differentially expressed ferroptosis genes in ASD samples and healthy controls. **(F)** Representative correlation plot of 16 differentially expressed FRGs. Blue represents positive correlation, and red represents positive correlation. The area of the pie chart represents the specific value of correlation coefficients. **(G)** Representative gene relationship network diagram of 16 differentially expressed FRGs.

The gene expression profiles of three GEO datasets (GSE18123, GSE42133, and GSE89594), including 201 control subjects and 293 ASD children, were obtained from the GEO database. The detailed flow chart is exhibited in Figure 1. A total of 18,242 genes (including 224 FRGs) were obtained when combined these datasets. Samples from these independent datasets presented distinct clusters before batch effect removal (Figure 2A) but clustered together after batch correction (Figure 2B). Next, we analyzed the expression profiles of 224 FRGs between the control and ASD groups, 16 of which were identified as the differentially expressed ferroptosis genes, including six upregulated and ten downregulated genes (Figures 2C–E). Spearman’s correlation analysis was performed to illuminate the correlation patterns between these FRGs (Figure 2F). Subsequently, these 16 FRGs were visualized through a gene relationship network diagram, which facilitated a comprehensive analysis of the interaction and interrelationship between genes (Figure 2G).

**FIGURE 3:**
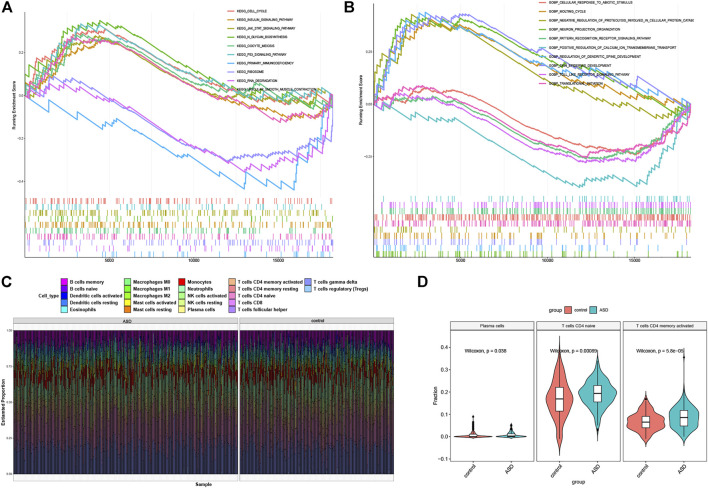
Functional enrichment and immune infiltration analysis between healthy controls and ASD children. **(A** and **B)** GSEA enrichment analysis showing significantly activated pathways **(A)** and biological functions **(B)** in ASD patients. **(C)** Representative barplot of the relative proportion of 22 infiltrated immune cells in control and ASD samples. **(D)** Box plots showing the heterogeneity of infiltrated immune cells between control and ASD.

To explore the potential enrichment pathways and biological functions between control and ASD subjects, we performed GSEA and found the pathways were mainly implicated in cell cycle, RNA processing, meiosis, classic signaling pathways, and immunodeficiency (Figure 3A). The enriched biological functions were mainly associated with the regulation of neural functions, the initiation of translation, regulation of protein catabolism, calcium ion balance, and immune response (Figure 3B). It suggests that the alterations in the immune system may be one of the primary pathogenesis involved in ASD progression. Therefore, we further analyzed the difference in the proportion of 22 immune cell subtypes between the two groups on the basis of the CIBERSORT algorithm (Figure 3C), and the results showed that the infiltration levels of plasma cells, naïve CD4 T cells, and activated CD4 memory T cells were significantly higher in ASD patients (Figure 3D). These findings revealed that the activation of the immune system was closely related to ASD progression.

### Identification of Ferroptosis Subtypes in Autism Spectrum Disorder

**FIGURE 4:**
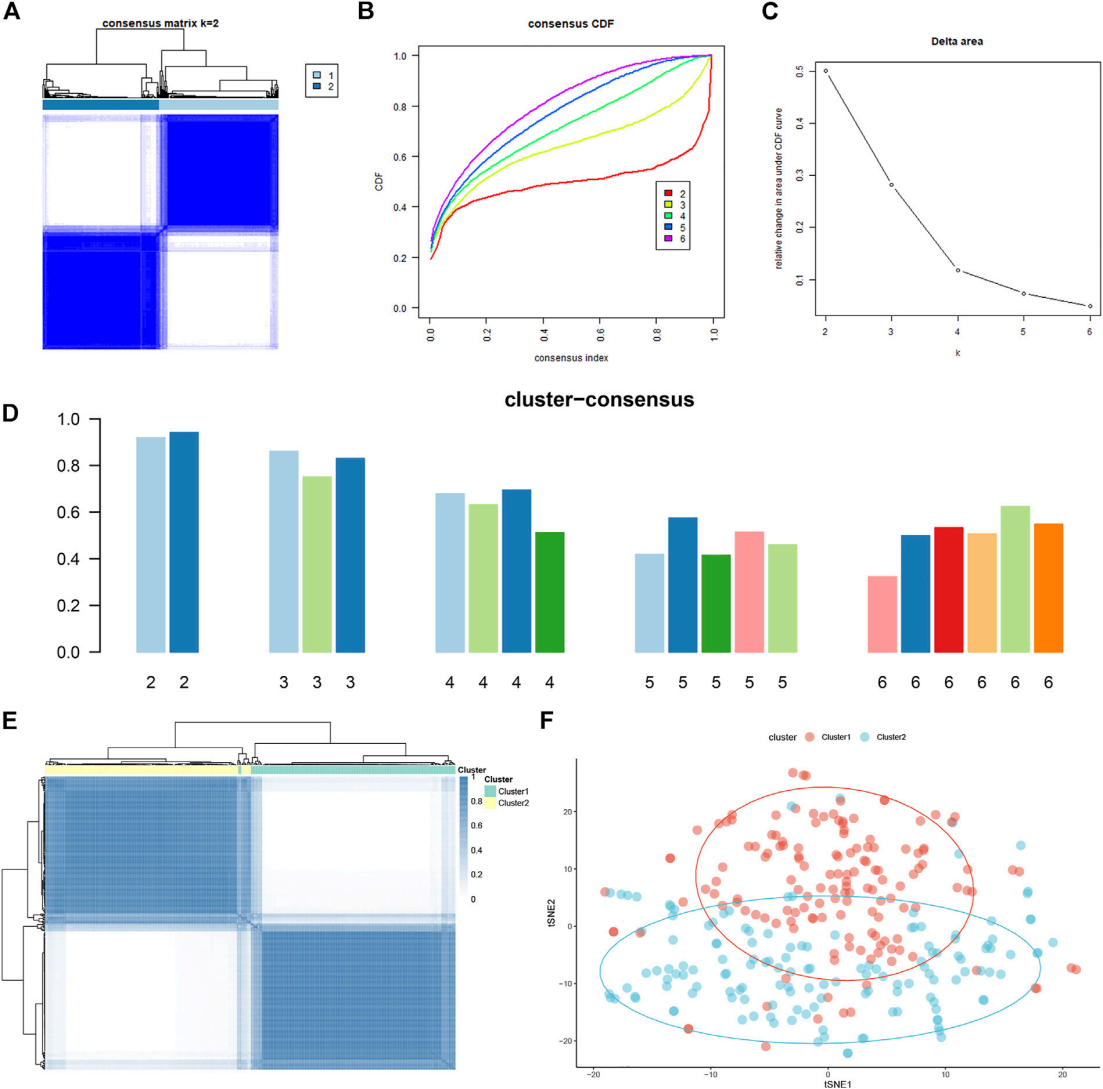
Identification of molecular clusters based on FRGs in ASD. **(A)** Consensus clustering matrix when k = 2. **(B–E)** Cumulative distribution function (CDF) curves of clustering **(B)**, CDF delta area curves **(C)**, consensus clustering score of each cluster **(D)**, and non-negative matrix heatmap **(E)** for verification of the clustering results. **(F)** t-SNE visualizes the distribution of two subtypes.

To elucidate the subtypes of ferroptosis in ASD, the expression profiles of 16 FRGs in 293 ASD samples were analyzed using the consistent unsupervised methodology. Clustering results showed that when the number was set to two (k = 2), the number of subtypes was most stable (Figure 4A). The fluctuation ranges of CDF curves are minimum at consensus index 0.2–0.6 when the value of k was set to two (k = 2) (Figure 4B). The changes in delta areas were presented in the CDF plots when k = 2–6 (Figure 4C). Moreover, the consensus score of each cluster was close to or more than 0.9 when k = 2 (Figure 4D). Combined with results of the consensus matrix, we clustered 293 ASD children into two subgroups, including Cluster1 (*n* = 148) and Cluster2 (*n* = 145) (Figure 4E). In addition, the results of principal component analysis (PCA) further confirmed that the two clusters were clearly distinguished (Figure 4F).

### Pathways Activity and Biological Function Between Ferroptosis Clusters

**FIGURE 5:**
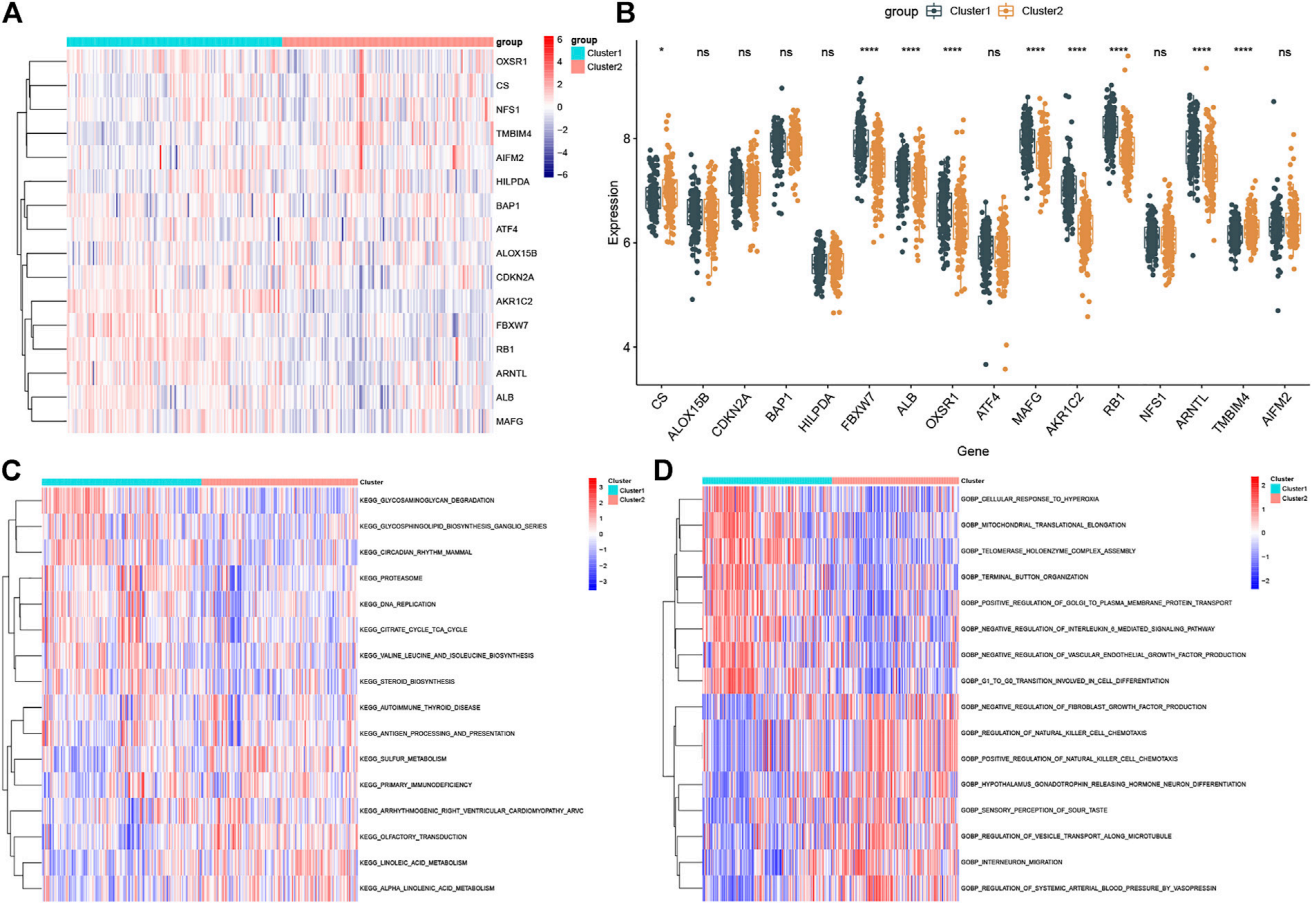
Biological characteristics between the two ferroptosis clusters. **(A** and **B)** Representative heatmap **(A)** and box plots **(B)** of 16 FRGs between the two ferroptosis clusters. *p < 0.05, ****p < 0.0001, ns, no significance. **(C** and **D)** GSVA analysis showing significantly activated pathways **(C)** and biological functions **(D)** in different ferroptosis clusters.

Compared to the expression profiles of 16 FRGs identified between control and ASD samples, a distinct expression pattern was generated between the two ferroptosis clusters (Figure 5A). Cluster1 exhibited higher expression levels of FBXW7, ALB, OXSR1, MAFG, AKR1C2, RB1, and ARNTL, while Cluster2 was characterized by enhanced expression of CS and TMBIM4 (Figure 5B). We further performed a GVSA analysis to evaluate the differences in pathways activity and biological functions between the two clusters. The results showed that TCA cycle, DNA replication, regulation of proteasome, and biosynthesis were upregulated in Cluster1 while metabolism-related signaling, olfactory transduction, and immune response were upregulated in Cluster2 (Figure 5C). Additionally, Cluster1 was mainly involved in the biological functions such as response to hyperoxia, translation and transportation of protein, IL-6 and VEGF mediated pathway, and cellar differentiation, while Cluster2 was closely associated with immune regulation, neuronal differentiation and migration, and transportation of microtubule (Figure 5D). These results revealed that the enriched pathways and biological functions are remarkably different between ferroptosis clusters of ASD patients. Distinct treatment strategies should be adopted for different subtypes.

### Characteristics of Immune Infiltration Between Ferroptosis Clusters

**FIGURE 6:**
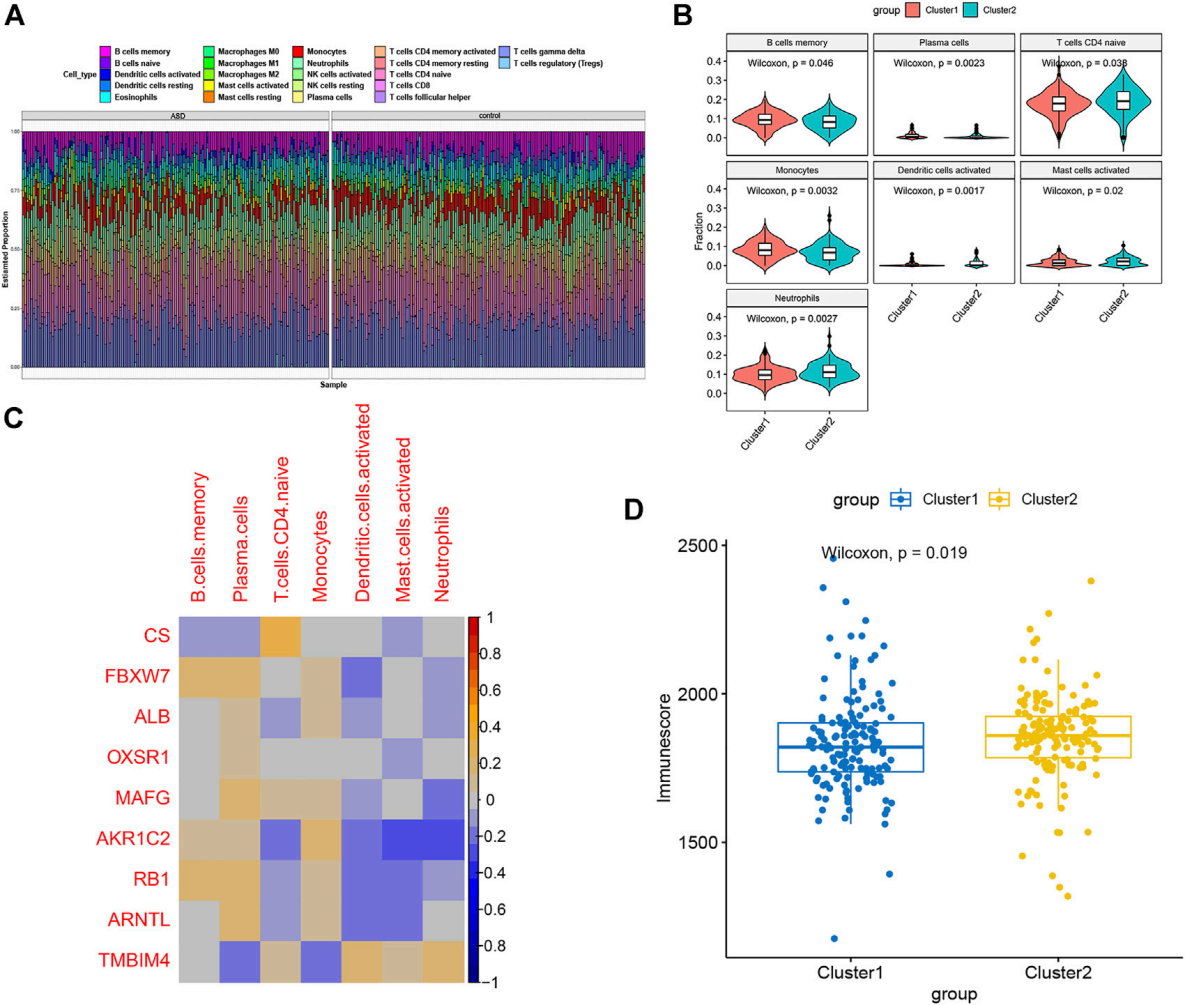
Characteristics of immune infiltration cells between ferroptosis subtypes. **(A)** The relative proportions of the 22 immune cell subclasses in all 293 ASD samples. **(B)** Box plots showing the heterogeneity of infiltrated immune cells between ferroptosis subtypes. **(C)** Representative correlation heatmap between differentially expressed immune cells and FRGs. **(D)** Estimated Immunoscore between the two ferroptosis subtypes.

To further evaluate the levels of immune cell infiltration between the two clusters, we utilized the CIBERSORT algorithm to calculate the relative proportion of the 22 immune cell subtypes in each patient sample (Figure 6A). The proportion of memory B cells, plasma cells, and monocytes was remarkably higher in Cluster1, while Cluser2 presented more abundance of naïve CD4 T cells, activated dendritic cells, activated mast cells, and neutrophils (Figure 6B). Recent studies have demonstrated that FRGs play a vital role in regulating immune responses and immune system function. Therefore, we carried out Spearman’s correlation analysis and found that CS and AKR1C2 were significantly correlated with immune cells (Figure 6C). Moreover, we comprehensively analyzed the immune score of two ferroptosis clusters and found that the Cluster2 group had a greater score than the Cluster1 group, which suggested that Cluster2 had an enhanced level of immune cell infiltration (Figure 6D).

### Comprehensive Analysis of DEGs Between Ferroptosis Clusters

**FIGURE 7:**
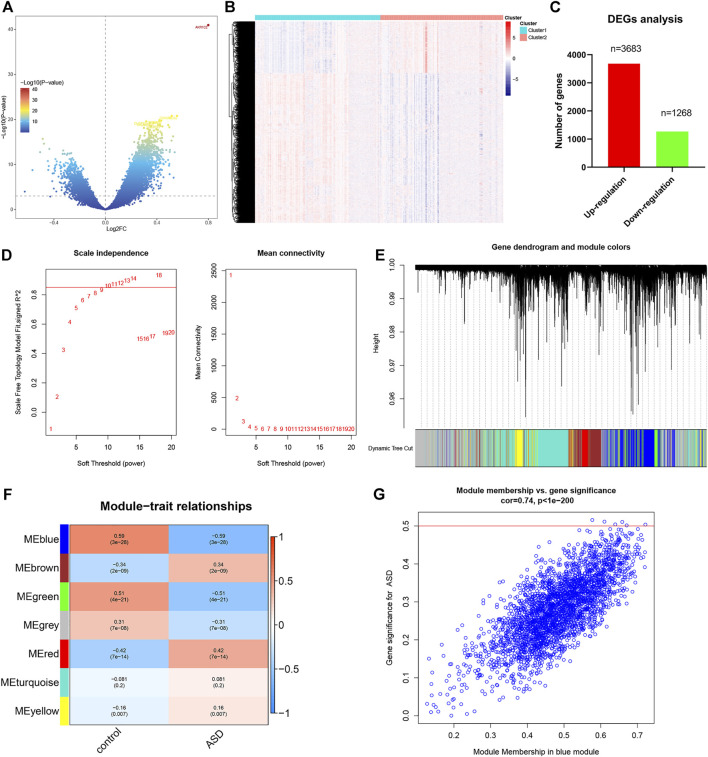
Identification of DEGs between ferroptosis subtypes. **(A** and **B)** Volcano plot **(A)** and heatmap **(B)** showing DEGs between ferroptosis subtypes. **(C)** Quantitative results of the number of upregulated and downregulated DEGs. **(D)** Analysis of scale-free fitting index and average connectivity for various soft-thresholding parameters. **(E)** Representative gene clustering dendrograms based on the distinct topological overlap and module colors. **(F)** Representative module-trait heatmap was established based on the eigenvalues values of the modules. **(G)** Representative scatter plot showing the correlation between the blue module and module-related genes.

In order to identify the DEGs related to ferroptosis clusters, we first carried out the DEGs method by using the R package of limma. A total of 4,951 DEGs were screened out, including 3683 DEGs with upregulation and 1268 DEGs with downregulation between Cluster1 and Cluster2 samples (Figures 7A–C). Next, on the basis of the whole gene expression profiles, the WGCNA algorithm was applied for constructing a co-expression network and potential modules most related to the ferroptosis subtypes. The optimal value soft threshold power was set to 10 to maintain a network with scale-free topology and efficient connectivity (Figure 7D). The cluster tree was clustered into seven modules with different colors by using the hierarchical clustering algorithm (Figure 7E). Among these modules, the blue module (2,973 genes) was most correlated with Cluster1 (R = 0.59) and Cluster2 (R = −0.59) (Figure 7F). Meanwhile, a remarkable correlation was presented between the blue module and module-related genes (cor = 0.74) (Figure 7G).

### Construction and Validation of the Ferroptosis Predictive Model

**FIGURE 8:**
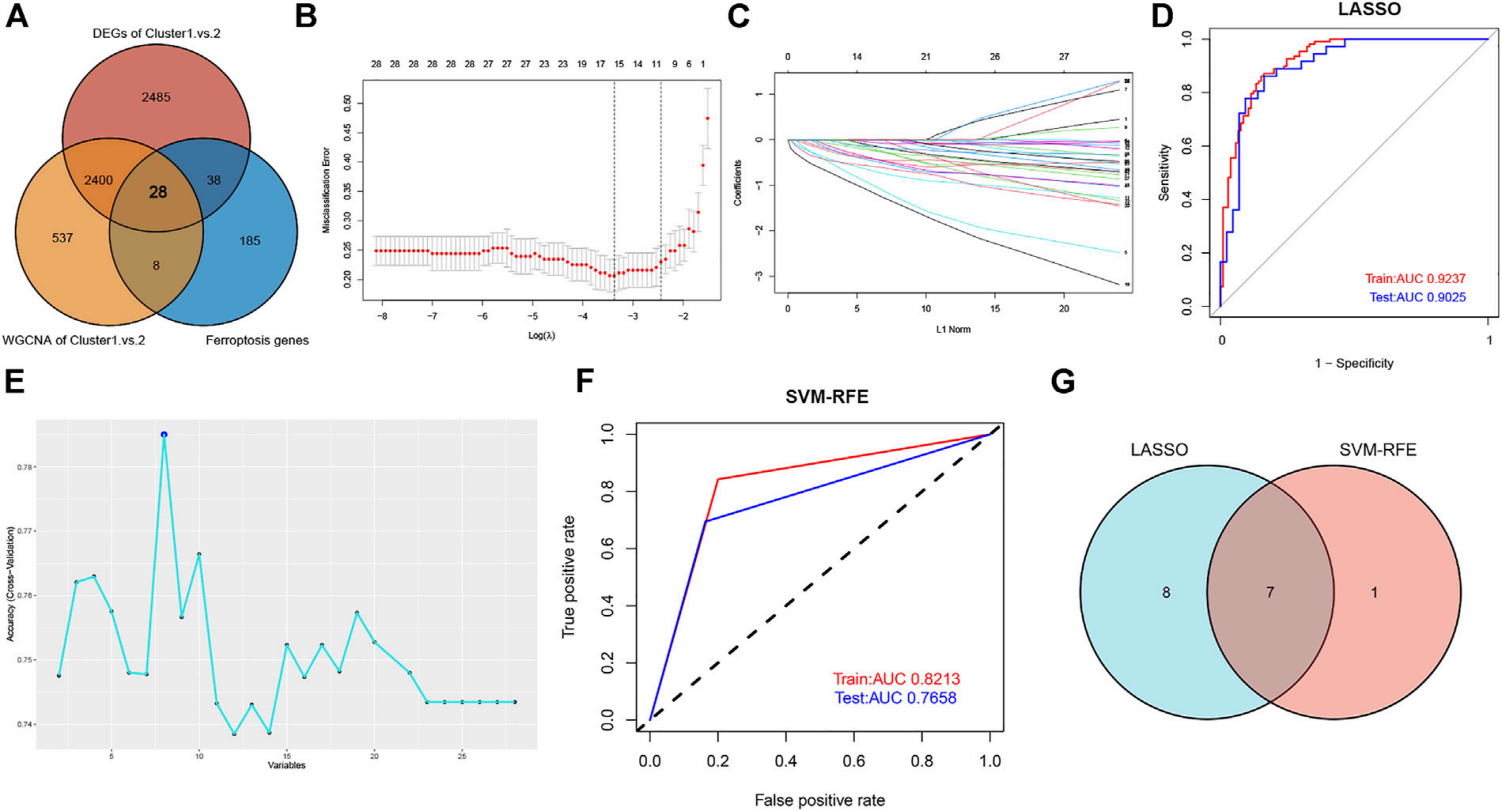
Construction of ferroptosis signature using machine learning algorithms. **(A)** Venn plot showing the co-expressional ferroptosis genes by intersecting the DEGs with the ferroptosis genes obtained from the FerrDb database. **(B)** The optimal lambda value was selected in the LASSO regression model based on 10-fold cross-validation. **(C)** The LASSO coefficient profiles of the 28 co-expressional ferroptosis genes. **(D)** Verification of the diagnostic value of the LASSO model in the training and testing sets by using ROC analysis. **(E)** The line graph showing the cross-validated accuracy based on different numbers of ferroptosis genes in the SVM-RFE model. **(F)** Verification of the diagnostic value of the SVM-RFE model in the training and testing sets by using ROC analysis. **(G)** Screening of seven key ferroptosis genes using LASSO and SVM-RFE machine learning algorithms.

A predictive model was constructed to further elucidate the role of ferroptosis in the heterogeneity of ASD patients. First, we intersected the DEGs identified by the DEGs and WGCNA algorithm with the ferroptosis genes obtained from the FerrDb database, yielding a total of 28 overlapping ferroptosis genes (Figure 8A). Next, the expression profiles of these ferroptosis genes were fit into LASSO logistic regression analysis on the basis of the least square method. The optimal value of lambda was demonstrated, and 15 potential key genes with nonzero coefficients in the training set were screened out (Figures 8B and C). Moreover, the value of AUC was plotted to estimate the capability of the LASSO logistic regression model in distinguishing Cluster1 from Cluster2, indicating that the AUC value was 0.9237 in the training set and 0.9025 in the validation set (Figure 8D). In addition, we also performed the SVM-RFE machine learning algorithm in the training set and identified eight effective predictors (Figure 8E). The AUC value of the SVM-RFE model in the training and testing sets was 0.8213 and 0.7658, respectively (Figure 8F), suggesting the accuracy of the model in predicting ASD subtypes. A total of seven ferroptosis-related predictive genes (RB1, ARNTL, FBXW7, LONP1, SESN2, AKR1C3, and JUN) were acquired by intersecting the two algorithms (Figure 8G).

**FIGURE 9:**
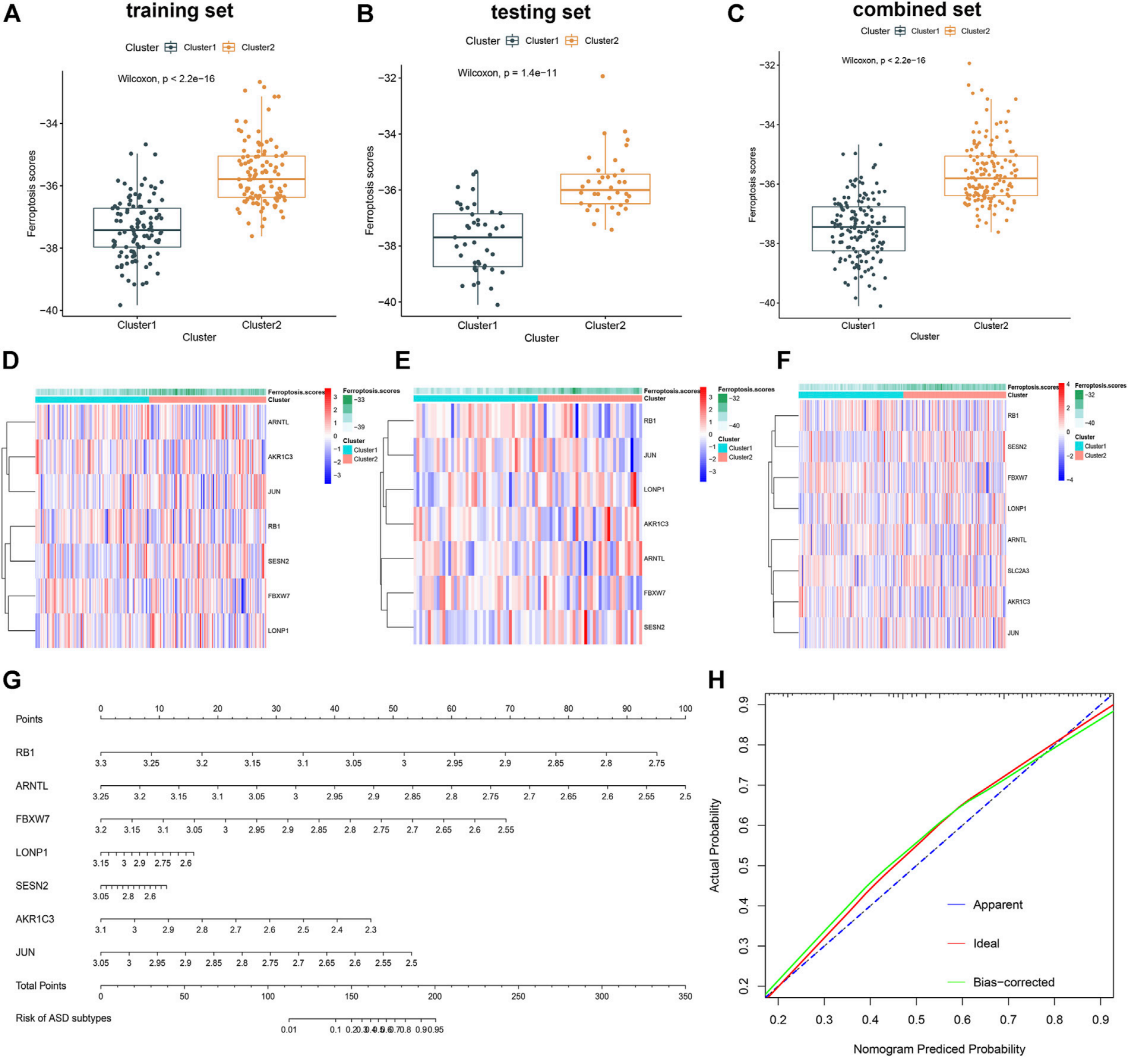
Construction and validation of the ferroptosis scores model. **(A–C)** Difference of ferroptosis scores between two ferroptosis clusters in the training **(A)**, testing **(B)**, and combination sets **(C)**. **(D–F)** Representative heatmap of the seven genes utilized to construct the ferroptosis scores in the training **(D)**, testing **(E)**, and combination sets **(F)**. **(G)** Representative nomogram for predicting the risk of ASD subtypes based on the 7 key FRGs. **(H)** Representative calibration curves for evaluating the predictive ability of the nomogram model.

Subsequently, a ferroptosis scores model was established as follows: ferroptosis scores = (−1.319092 × RB1) + (−1.175048 × ARNTL) + (−0.763953 × FBXW7) + (−0.305409 × LONP1) + (−0.051775 × SESN2) + (−0.541050 × AKR1C3) + (−0.634476 × JUN). We, therefore, further analyzed the association between ferroptosis scores and ferroptosis subtypes in the training, testing, and combination sets. Cluster1 was remarkably related to the low ferroptosis scores, while Cluster2 exhibited the relative higher scores of ferroptosis, which indicate that the high scores of ferroptosis may be closely correlated with a greater level of immune cell infiltration (Figures 9A–C). Meanwhile, the plotted heatmaps indicated that the expression levels of these seven ferroptosis scores–related genes differed significantly between the Cluster1 and Cluster2 ASD patient groups in training, testing, and combination sets (Figures 9D–F). Considering the precise predictive capability of the ferroptosis scores model, we constructed a nomogram to evaluate the risk of ASD subtypes more clearly (Figure 9G). The calibration curves of the nomogram also demonstrated the accurate prediction (Figure 9H).

## Discussion

Due to the apparent heterogeneity of the prognosis and clinical symptoms in ASD children, the overall treatment outcomes of ASD are highly limited (Masi et al., 2017). Promising medical therapies, including behavioral interventions and antipsychotic medications, have been implicated in the treatment of ASD over the past decades. However, drug resistance against ASD may occur frequently if traditional classifications based on gender and comorbidities are used to guide anti-ASD treatment (Anderson et al., 2007; Ospina et al., 2008; Woolfenden et al., 2012; Aslam and Altaf, 2019; 2021). Therefore, accurate differentiation of ASD clusters at the molecular level will improve the understanding of the heterogeneity in ASD and is vital to guide the individualized treatment of ASD.

Ferroptosis is a recently reported novel mode of Fe^2+^-dependent cell death characterized by intracellular oxidative stress–induced lipid peroxide overload. An increasing number of studies have demonstrated that ferroptosis plays a critical role in the progression of diseases, including tumors and non-tumors (Louandre et al., 2015; Xie et al., 2017; Li et al., 2020). At present, several primarily pathways have been implicated in the regulation of ferroptosis: First, the inhibition of system Xc^−^ has been found to affect the synthesis of GSH and GPX4 by suppressing the absorption of cysteine, which, in turn, results in the decline of antioxidants activity and the accumulation of lipid peroxides, eventually leading to the occurrence of ferroptosis (Jiang et al., 2015a; Jiang et al., 2015b). In addition, the ferritin metabolism-related pathways such as ATG5-ATG7-NCOA4 and p62-Keap1-NRF2 axis are considered critical in regulating the metabolism of intracellular Fe^2+^ and the formation of ROS, thus having a regulatory role in ferroptosis (Hou et al., 2016; Sun et al., 2016). Furthermore, ACSL4 and P53-SAT1-ALOX15-dependent lipid metabolism pathways are closely associated with the biosynthesis of phosphatidylethanolamine (PE) and polyunsaturated fatty acids (PUFAs), which, in turn, promote the activation of ferroptosis (Yang and Stockwell, 2016; Kagan et al., 2017). Although the relationship between ferroptosis and disease is increasingly demonstrated, whether ferroptosis can contribute to the progression of ASD and the potential molecular mechanisms of ferroptosis in ASD remains unclear. Therefore, identifying the potential mechanisms of ferroptosis and illustrating the ferroptosis-related ASD subtypes is badly needed.

In the current study, we first systematically analyzed the expression profiles of genes related to ferroptosis between normal and ASD samples. A total of 16 FRGs in ASD patients were distinct from those in normal samples, suggesting that ferroptosis might play a critical role in the pathogenesis of ASD. Subsequently, the correlation among ferroptosis regulators was evaluated to clarify the complex relationships among these regulators in children with ASD and healthy individuals. Additionally, we explored the altered levels of infiltrated immune cells and found that ASD children exhibited higher levels of plasma cells and T cells, which was consistent with previous studies (Meltzer and Van de Water, 2017; Uddin et al., 2020). Furthermore, two distinct subtypes were classified based on the expression of 16 genes related to ferroptosis. GSVA enrichment analysis indicated that cluster1 was primarily enriched in pathways associated with DNA replication, proteasome biosynthesis, and response to stress. Cluster2 was prominently associated with metabolism-related signaling and immune regulation. Presently, increasing researches have proven that ferroptosis is closely related to immune response (Stockwell and Jiang, 2019; Wang et al., 2019; Tang et al., 2020). For example, the accumulation of lipid peroxides is usually accompanied by a higher expression level of CD8+T cells, thus, enhancing the anti-tumor immune efficacy (Wang et al., 2019). Meanwhile, the activation of ferroptosis plays a vital role in prompting the differentiation of B cells and natural killer cells by suppressing bone morphogenetic protein (BMP) (Chen X. et al., 2021). Therefore, we further examined the association between two ferroptosis clusters and infiltrated immune cells. In our current study, Cluster1 presented activated memory B cells, plasma cells, and monocytes, while in Cluster 2, the most remarkable immune infiltrating cells were naïve CD4 T cells, activated dendritic cells, activated mast cells, and neutrophils. Neutrophils and activated mast cells are considered key factors in the occurrence and progression of various diseases (Levick and Widiapradja, 2018; Chen F. et al., 2021). Dendritic cells (DCs), the most important antigen-presenting cells, play a primary role in regulating the adaptive immune response by identifying, processing, and presenting external antigens to T cells (Song et al., 2018). Activated DCs have been reported to be closely associated with inflammatory disease processes (Wilensky et al., 2014). Moreover, Cluster2 patients also exhibited a higher immune score. Therefore, these results suggest patients with ferroptosis Cluster2 are related to enhanced immune response and represent the worse ASD prognosis. The reason for the remarkable difference in infiltrated immune cells between the two ferroptosis clusters may be due to the fact that ferroptosis cells have the ability to absorb immune cells to their location by releasing amino acid oxidation products, ultimately causing immune activation (Friedmann Angeli et al., 2019). However, the molecular mechanism between ferroptosis and immunity remains unclear.

Considering that ferroptosis may be the key regulator responsible for the individual heterogeneity of ASD, we, therefore, constructed a predictive model based on 28 co-expressional FRGs to quantify ferroptosis scores. We constructed a ferroptosis score model with seven identified ferroptosis genes (RB1, ARNTL, FBXW7, LONP1, SESN2, AKR1C3, and JUN) to predict the subtypes of patients who had ASD. RB1 and FBXW7 are the classical tumor suppressor genes, and their deletion or inactivation has been reported to be implicated in tumorigenesis (Dyson, 2016; Yeh et al., 2018). ARNTL is a core molecule involved in the circadian clock loop and plays a critical role in regulating physiological and complex circadian rhythms. Meanwhile, ARNTL is essential to promote the development of hard tissues such as bones, cartilage, and teeth. ARNTL deficiency affects bone development and disrupts the rhythmic cycle of monocytes, thus leading to the occurrence of inflammation-related diseases (Nguyen et al., 2013; Chen et al., 2020). Studies have reported that LONP1, a novel mitochondrial protease, is involved in degrading misfolded or damaged proteins and regulating mitochondrial quality control (Gibellini et al., 2020). SESN2 act as an effective anti-oxidant to prevent the progression of neurological and immune system–related diseases (Wang et al., 2021). Bioinformatics analysis has proven that AKR1C3 serves as a promising biomarker for acute myocardial infarction (Liang et al., 2021). Additionally, studies have demonstrated that activation of JUN is related to the poor prognosis of various diseases (Schenkel, 2004). Consistently, children in Cluster2 had higher ferroptosis scores than children in Cluster1, and the model exhibited a high value of AUC both in the training and testing sets. Moreover, the constructed nomogram was utilized to predict the occurrence of ASD subtypes, and the calibration curves also indicated that the predictive model had a high accuracy to quantify the ASD subtypes.

Some limitations must be clarified in our study. First, our study lacked additional clinical characteristics, including age, gender, and childhood autism rating scale (CARS) to further validate the performance of the ferroptosis scores model in predicting the ASD clusters. Additionally, further studies should collect more prognostic information to assess the prognostic value of the ferroptosis scores model in different ASD subtypes. Furthermore, larger numbers of ASD samples need to be taken into account to validate the stability of the subgroups, and the detailed mechanism between ferroptosis and immunity requires further verification.

## Conclusion

In summary, this study found two ferroptosis-related clusters in ASD children and clarified the discrepant immune infiltration cells between ASD children with different ferroptosis subtypes. Predictive models constructed based on LASSO and SVM-RFE algorithms proved to be of high diagnostic value. Importantly, the ferroptosis scores model based on seven signatures has the ability to accurately distinguish the molecular subtypes of children with ASD, which may provide novel insights to explore the pathophysiology of clinical presentation and prognostic heterogeneity in children with ASD.

## Data Availability

The datasets presented in this study can be found in online repositories. The names of the repository/repositories and accession number(s) can be found in the article/Supplementary Material.

## Abbreviations

ASD, autism spectrum disorder; AUC, area under the ROC curve; DEGs, differentially expressed genes; FRGs, ferroptosis-related genes; GEO, Gene Expression Omnibus; GO, Gene Ontology; GSEA, Gene Set Enrichment Analysis; GSVA, Gene Set Variation Analysis; KEGG, Kyoto Encyclopedia of Genes and Genomes; LASSO, least absolute shrinkage and selection operator regression; ROC, receiver operating characteristic; SVM-RFE, recursive feature elimination for support vector machines; WGCNA, weighted gene co-expression network analysis.
